# Transgelin Up-Regulation in Obstructive Nephropathy

**DOI:** 10.1371/journal.pone.0066887

**Published:** 2013-06-26

**Authors:** Fani Karagianni, Niki Prakoura, Garyfallia Kaltsa, Panagiotis Politis, Elena Arvaniti, Valeria Kaltezioti, Stelios Psarras, Stamatis Pagakis, Michalis Katsimboulas, Ahmed Abed, Christos Chatziantoniou, Aristidis Charonis

**Affiliations:** 1 Biomedical Research Foundation of the Academy of Athens, Section of Histology, Center for Basic Research I, Athens, Greece; 2 Biomedical Research Foundation of the Academy of Athens, Biological Imaging Unit, Athens, Greece; 3 Biomedical Research Foundation of the Academy of Athens, Center for Experimental Surgery, Athens, Greece; 4 INSERM and Université Pierre et Marie Curie-Paris VI, Paris, France; University of Pittsburgh, United States of America

## Abstract

Fibrosis is a complex and multifactorial process, affecting the structure and compromising the function of several organs. Among those, renal fibrosis is an important pathological change, eventually leading to renal failure. Proteomic analysis of the renal parenchyma in the well-established rat model of unilateral ureteral obstruction (UUO model) suggested that transgelin was up-regulated during the development of fibrosis. Transgelin up-regulation was confirmed both at the protein and at the mRNA level. It was observed that at early stages of fibrosis transgelin was mainly expressed in the interstitial compartment and, more specifically, in cells surrounding the glomeruli. Subsequently, it was confirmed that transgelin expressing cells were activated fibroblasts, based on their extensive co-expression of α-SMA and their complete lack of co-distribution with markers of other cell types (endothelial, epithelial and cells of the immune system). These periglomerular fibroblasts exhibited staining for transgelin mainly cytoplasmic but occasionally nuclear as well. In addition, transgelin expression in periglomerular fibroblasts was absent in renal fibrosis developed in a hypertensive model, compared to the UUO model. Promoter analysis indicated that there are several conserved motifs for transcription factor binding. Among those, Kruppel-like factor 6 was found to be up-regulated in transgelin positive periglomerular activated fibroblasts, suggesting a possible involvement in the mechanism of transgelin up-regulation. These data strongly suggest that transgelin is up-regulated in the obstructive nephropathy and could be used as a novel marker for renal fibrosis in the future.

## Introduction

It has recently being realized that Chronic Kidney Disease (CKD) is a condition detected in high frequency in all adult populations [1]. The most common anatomical finding in CKD, irrelevant of its etiology, is renal fibrosis. Therefore understanding the molecular mechanisms operating during the fibrotic procedure and finding early markers for its detection is of great importance.

Among the macromolecules involved in renal pathology in diseases leading to CKD is transgelin, also known as SM22; it is a smooth muscle cytoskeletal protein interacting with actin [2]. Microarray analysis [3], [4] and proteomic analysis [5] have provided evidence for the up-regulation of transgelin in animal models of renal fibrosis. Most recent studies have focused on the up-regulation of transgelin in glomerular diseases [6], [7], [8], [9] and have demonstrated that the cell type where transgelin is mainly upregulated can vary (glomerular parietal, glomerular visceral, but also tubular interstitial cells) depending on the etiology of the disease and the time point of tissue collection. Furthermore, work from our group in biopsy material from several patients with glomerular renal diseases has demonstrated a wide range of cells involved in transgelin up-regulation [10].

However, no studies exist for the expression of transgelin in obstructive renal diseases. Obstructive nephropathy is the main cause of CKD in children [11] and the animal model of Unilateral Ureteric Obstruction (UUO) is an ideal model for this condition [12]. Therefore, in this report, we use the animal model of UUO to study the expression of transgelin. First, we provide data from proteomic analysis as well as biochemical and morphological approaches to document the alterations in the expression of transgelin. Second, we focus on the specific cell type that demonstrates up-regulated expression and by dual immunofluorescence using appropriate markers we characterize this cell type as a periglomerular fibroblast. And third, we compare transgelin up-regulation in another animal model of renal fibrosis, induced by elevated blood pressure. Therefore, our data suggest that transgelin is up-regulated in the obstructive nephropathy and consequently could be used as a future novel marker for renal fibrosis.

## Materials and Methods

### UUO Model

Studies were performed on male Wistar rats weighing between 200 and 250g, supplied from the colony of the Center of Experimental Surgery of our Institute. The rats were maintained on a standard rodent diet with free access to water. During the entire experiment rats were kept in individual cages with a 12-h artificial light–dark cycle at 21±1°C. The rats were under the constant care of the Division of Animal Facility of our Institute. Before surgery the rats were anesthetized with intraperitoneal injection of 80 mg/kg body weight of ketamine and 10 mg/kg body weight of xylosine. The animals were divided into four groups: 8 operated, and 8 sham-operated sacrificed 2 days after ligation, 8 operated and 8 sham-operated sacrificed 8 days after ligation. The right ureter was exposed through a midline abdominal incision and was either completely obstructed 1 cm below the renal pelvis with 5.0 silk ligature (operated animals) or manipulated similarly but not ligated (sham-operated animals). The midline incision was closed; the animals were allowed to recover from the anesthesia and placed back into their cages. Aseptic conditions were kept during the whole surgery. Two and eight days after surgery the animals were anesthetized similarly and their kidneys were collected. All the kidneys were thoroughly rinsed with isotonic saline containing protease inhibitors (1 tablet/10 mL, Roche Diagnostics), the area of the cortex was dissected into small pieces and then stored either in 10% formaldehyde for morphological studies or in liquid nitrogen for biochemical analysis. At the end of the experiment all animals were sacrificed by exsanguination. The same procedure was performed for mouse (C57BL/6) UUO. All animal procedures were approved by the Animal Experimentation Committee of the Greek Government.

### Hypertensive Model

Experiments were performed using a transgenic strain of mouse (RenTg) backcrossed in the genetic background 129SV that is described elsewhere [13]. Briefly, RenTg mice express a rennin transgene inserted into a liver-specific locus and driven by a liver-specific promoter/enhancer. The rennin coding sequence (Ren2/1d) is a synthetic cDNA consisting of parts of the Ren-2 and Ren-1d genes modified to include glycosylation sites for increased stability, a furin cleavage site to enable prorennin to active rennin processing to occur in the liver and allow secretion of active rennin into the blood stream. Thus, this transgenic strain expresses rennin ectopically at a constant high level in the liver and leads to elevated mRNA and protein levels of prorennin and active rennin. In our case, this model was produced in Dr. Chatziantoniou’s lab according to the above procedure [14].

The time points that they were used for this model were the 9, 12, 21 and 53 weeks.

In all three animal models used (rat and mouse UUO and hypertensive model), the extent of fibrosis has been quantitated by Sirius Red staining as described [5], [15], [16]. For the RenTG model, the time intervals selected for double immunofluorescence (12 weeks and 52 weeks) correspond to the fold- increase of Sirius Red staining observed at the 2-day and 8-day interval of the rat UUO model.

### Immunohistochemistry

Transgelin expression was also examined using an immunoperoxidase staining technique on paraffin embedded tissue specimens. The tissue specimens were derived from the UUO model. Tissue sections (5 µm) were deparaffinized and rehydrated. Endogenous peroxidase was inactivated with 3% H_2_O_2_ in methanol for 30 min at room temperature in the dark. The sections were incubated for 30 min at room temperature with blocking solution containing purified rat IgG diluted in PBS-0.1% Triton X-100. For negative controls tissue sections were incubated with either only the secondary antibody or non-specific rabbit IgG in a similar concentration with that of anti-transgelin antibody. For immunohistochemical staining, we used the anti-transgelin 1 Abcam antibody [Ab (14106)]. This is a polyclonal antibody raised in rabbit against amino acid sequence 189–200 of mouse transgelin 1; since transgelin 2 contains the exact same sequence, this antibody is expected to cross-react with transgelin 2. Sections were incubated at a concentration of 1 µg/ml for one overnight at 4°C. Both negative controls and experimental sections washed and finally incubated with goat anti-rabbit-HRP specific antibody (Sigma) for 60 min at room temperature. The detection reaction was developed with 30, 30-diaminobenzidine (Vector Laboratories). Nuclei were counterstained with haematoxylin before examination. All tissue sections were dehydrated in graded alcohols and xylene and embedded in mounting solution (DPX).

### Double Immunofluorescence

Double immunofluorescence was carried in UUO model using transgelin and a variety of other cell type markers in order to confirm co-localization. Additionally, double immunofluorescence experiments were performed in the hypertensive model for transgelin and KLF6. Tissue sections (5 µm) were deparaffinized and rehydrated. For negative controls tissue sections were incubated with only the secondary antibody. The sections were incubated for 30 min at room temperature in the dark with blocking solution containing purified rat IgG diluted in PBS-0.1% Triton X-100. Then, the sections were incubated with the rabbit polyclonal anti-transgelin antibody with characteristics as described above, in concentration of 1 µg/ml for one overnight at 4°C and aSMA (abcam76549), RECA-1 (ab9774), galectin-3 (Santa Cruz (N20) ), CD11b (BD Pharmingen (553307), Fsp1 (abcam 93283), KLF-6 (Santa Cruz (sc-7158) in the same concentration. Both for negative controls (sections incubated only with the secondary antibody) and experimental sections washed and finally incubated with Cy-2 anti-rabbit and Cy-3 anti-mouse antibody for 60 min at room temperature in the dark. All the sections washed with PBS and then incubated with DAPI for 10 min in the dark. All tissue sections were then mounted with moviol.

### Western Blotting

Transgelin expression was assayed by immunoblot analysis. Tissue samples were homogenized and proteins were extracted with sample buffer (50 mM Tris-HCl, 1% NP-40, 0.25% deoxycholate, 150 mM NaCl, 1 mM Na_2_EDTA, 1 mM fluoride, 10 mL of a cocktail of protease inhibitors (Sigma) for 0.2 g of tissue). Equal amounts of protein from each sample (50 mg of protein) were run on 12% polyacrylamide gels and transferred to a Nitrocellulose membrane. Membrane was pretreated with 5% skimmed milk in PBS for 60 min at room temperature and then probed with antibodies against transgelin (Abcam 14106) at a concentration of 1 µg/ml in 1% blocking solution at 4°C, with constant agitation overnight. The bound antibody was labelled with horseradish peroxidase-conjugated secondary antibody (Sigma) for one and a half hour at room temperature with constant agitation and was detected with ECL (Perkin Elmer) detection system. Western blots were scanned with GS-800 calibrated densitometer and images were analyzed with Quantity One Image processing software (BioRad). b-tubulin was used to verify all protein loads and data were normalized to b-tubulin load, using a primary anti-b-tubulin antibody (Sigma– T0198).

### RNA Isolation, cDNA Synthesis and Quantitative Real Time-PCR

Tissue samples from sham operated, ligated for 2 days and ligated for 8 days animals (three animals from each group) were homogenized and total RNA was isolated using a Nucleospin kit (Macherey-Nagel). Two micrograms of DNase I treated RNA was used to synthesize cDNA with Super-Script™ II reverse transcriptase (Invitrogen) according to the manufacturer’s instructions. Quantitative real time PCR assays were done in triplicates using the SYBR GreenER kit of Invitrogen on the Chromo4 instrument (BioRad) in conjunction with gene-specific forward and reverse primers. Oligonucleotide primers were designed to amplify 140bp of rat transgelin 1: forward, 5′CAAGCAGATGGAACAGGTGGC3′; reverse, 5′GGACTGTAATGGCTTTGGGCA3′, 186bp of rat transgelin 2: forward, 5′CTTCCAGAACTGGCTCAAGG3′; reverse, 5′ GTCTGGAAGATGTCCGTGGT 3′. Primers were also designed to amplify 105bp from 18SrRNA gene: forward, 5′AACTTTCGATGGTAGTCGCCG3′; reverse, 5′CCTTGGATGTGGTAGCCGTTT3′. Cycle conditions for rat transgelin 1, transgelin 2 and 18S rRNA consisted of 52°C for 5 min, 95°C for 2 min, 60 cycles of 95°C for 15 s, 56°C for 40 s (plate reading), followed by melting curve analysis, from 55°C to 99°C with measurements taken every 0.5°C. The cycle thresholds (Cts) determined for transgelin 1, transgelin 2 mRNA were normalized to those of 18S rRNA to compensate for variability in RNA amount loaded in the RTstep. Quantification of the levels of transgelin 1 and transgelin 2 mRNA expressions was done with the DDCt method. Data were expressed as the ratio of transgelin 1 and transgelin 2 to the reference gene (18SrRNA).

### RT-PCR in the Hypertentensive Model

Primers were designed to amplify: 122bp of mouse transgelin 1, forward, 5′ AAGCCTTCTCTGCCTCAACAT 3′, reverse, 5′ CAATCCACTCCACTAGTCGCT 3′, 103bp of mouse transgelin 2, forward, 5′ TGTGGATCTCTGGGAAGGAAAG 3′, reverse, 5′ ATCCCCAGAGAAGAGCCCAT 3′. Primers were also designed to amplify 116bp of hypoxanthine guanine phosphoribosyl transferase (Hprt) gene, forward, 5′GGAGCGGTAGCACCTCCT 3′, reverse, 5′ CTGGTTCATCATCGCTAATCAC 3′.

Data were expressed as the ratio of transgelin 1 and transgelin 2 to the reference gene (HPRT) in the hypertensive model at variable time points (9, 12, 21 and 53 weeks) both in wild-type and hypertensive animals.

### Detection of Nuclear Localization of Transgelin

In order to ensure that transgelin is present in the nuclei of many periglomerular cells the following procedure took place in many glomeruli. Many pictures of selected glomeruli on the confocal microscope with a 63X/1.4NA lens were taken. Transgelin was not present in the negative control sections (data shown in File S1). We have used this lens in order to achieve high magnification but, more importantly, the thinnest optical section possible on our confocal microscope (0.7 µm with the pinhole set at Airy = 1). Since an average nucleus is at least 5 microns thick, we were thus able to "dissect" the nucleus at several 0.7 µm thick optical sections. In addition, using a small focus step of only 0.3 µm, we were also able to position the optical section with high accuracy in the middle of the nucleus. Thus we were certain that indeed several optical sections were going through the middle of the nuclei and did not contain any cytoplasmic layer above or below the nucleus. Therefore, if in these mid sections we could also see the chromophore of the transgelin antibody, we could then unequivocally reach the conclusion that the transgelin antibody chromophore was colocalised with the DAPI stain and hence was located inside the nucleus.

### Bioinformatic Analysis of Transgelin Promoter

The Genomatix software tools were used for the promoter analysis of transgelin. According to genomatix, for each gene a set of human, mouse, and rat orthologous promoters were extracted from genomic sequences. Transcription factor (TF) binding site analysis combined with a literature search was performed using ElDorado and Gene2Promoter tools. Therefore, promoter analysis of transgelin gave rise to many transcription factors that they have binding sites on transgelin promoter.

### Image J: Fiji Analysis

Fiji is a distribution of the popular open-source software ImageJ focused on biological-image analysis. This software is powerful and one of its useful tools is that of colocalization. The colocalization analysis implements and performs the pixel intensity correlation over space methods of Pearson, Manders, Costes, Li and more, for scatterplots, analysis, automatic thresholding and statistical significance testing. In order to perform the quantification of codistribution analysis, this tool was used as it is described on http://fiji.sc/Colocalization_Analysis.

### Statistical Analysis

All the results are presented as mean ± SD. To analyze the difference between two data sets, two-tailed student’s t-test was used. A p value less than 0.05 was considered significant. The p values are indicated in the figure legends. Asterisks indicate comparison of the condition with the control.

## Results

### Identification of Transgelin as a Differentially Expressed Protein

**Figure 1:**
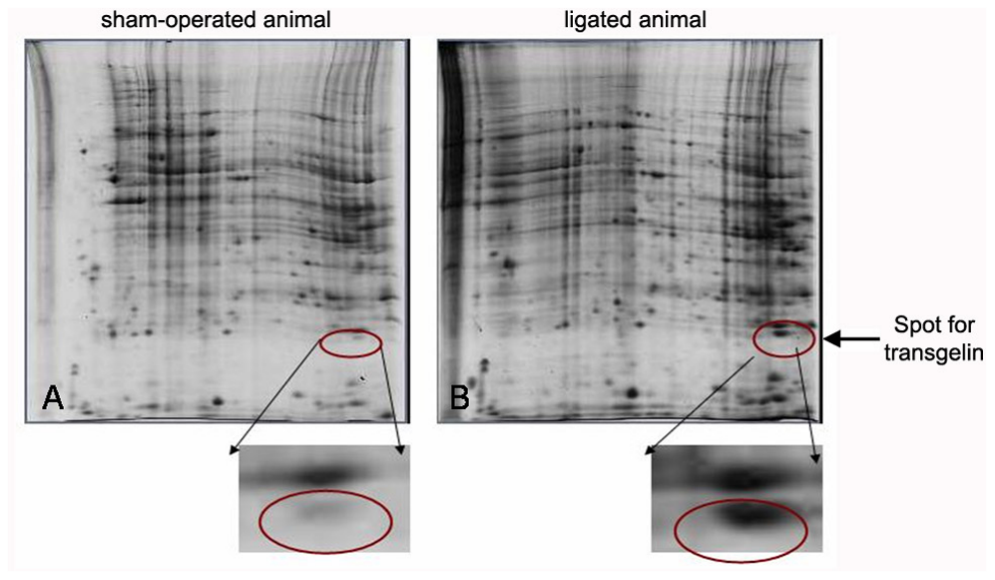
Proteomic analysis in rat UUO model. 2D gel electrophoresis from proteomic analysis of sham-operated (A) and ligated animals (B) sacrificed 8 days after ligation.

In the past, we have performed proteomic analysis in the UUO model in rats. These studies have generated tables of differentially expressed proteins during the development of fibrosis in this model and are published in a previous report [5]; the detailed methodology followed can be found in File S1 of the present work. Transgelin appeared as an upregulated protein [5]. In the current report we present the actual data on transgelin in 2-D gel electrophoresis. In figure 1 two representative 2-dimensional gels from sham-operated and ligated animal sacrificed at 8 days after ligation are shown; the inserts show in magnification the area where the spot corresponding to transgelin is located. It is clear that transgelin is up-regulated in the renal parenchyma following 8 days of ureteral ligation.

### Confirmation of Transgelin Up-regulation

**Figure 2:**
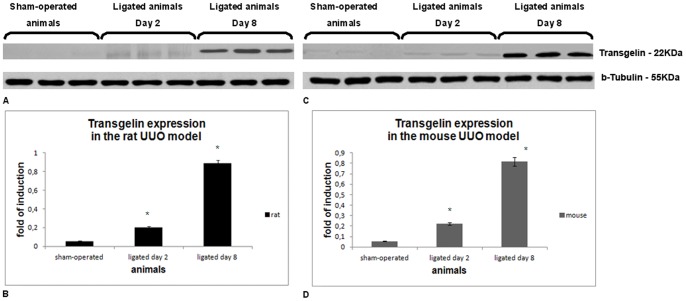
The expression of transgelin in the UUO model at the protein level. A) Western Blot Analysis of transgelin in sham-operated and ligated animals sacrificed 2 and 8 days after ligation in rats. B) Quantification of Western Blot Analysis in rat UUO model. C) Western Blot Analysis of transgelin in sham-operated and ligated animals sacrificed 2 and 8 days after ligation in mice. D) Quantification of Western Blot Analysis in mice UUO model. In B and D, the asterisk denotes a p value <0.05.

In order to confirm the proteomic data, we performed Western blot analysis, using sham-operated animals and animals sacrificed after 2-days or 8-days of ureteral ligation both in rats and mice. The results are shown in figure 2 on blots (Figure 2A & 2C) and following quantification of densitometric analysis (Figure 2B & 2D). In Figure 2A and 2C, 3 animals have been included per time point whereas in Figure 2B and 2D the quantification is calculated by the average of 5 different animals per time point. Sham-operated animals do express transgelin at a low level in both species; this is expected since transgelin is always expressed in smooth muscle cells existing in the vascular wall. However, it is clear that there is a slight increase in transgelin expression at the 2-day interval which becomes far more pronounced at the 8-day interval both in rats and mice. Both increases reached statistical significance.

**Figure 3:**
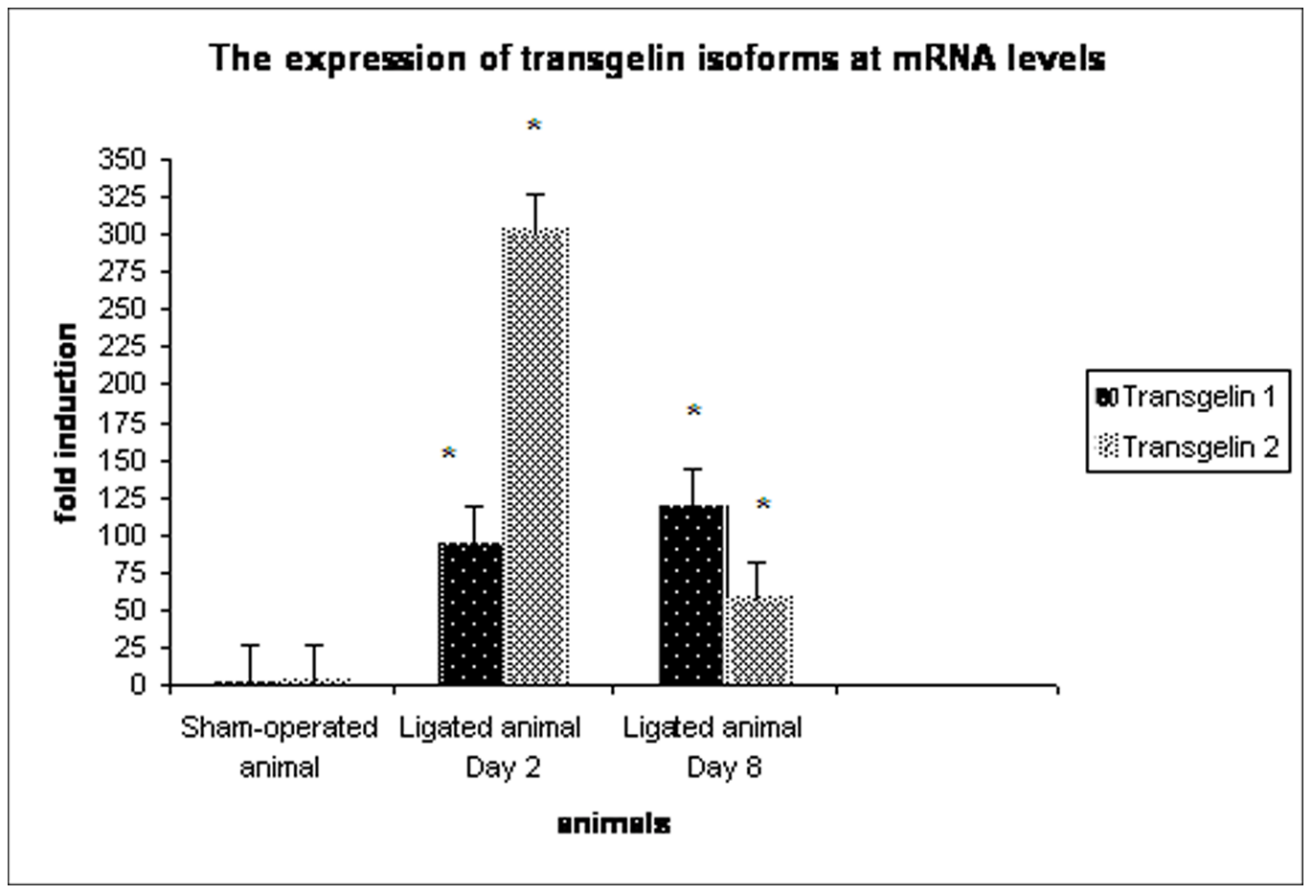
The expression of transgelin isoforms at the mRNA level. The expression of transgelin 1 and transgelin 2 *(p = 0.043)* is regulated at transcriptional level already from the early interval. The asterisk indicates a p value <0.05.

In order to explore whether the up-regulation of transgelin also occurs at the transcriptional level, we performed real-time PCR analysis, using appropriate primers in the UUO model. It is known that there are three different transgelin isoforms, each encoded by a different gene. Since in the kidney there are only two isoforms expressed, transgelin 1 (also known as SM22a) and transgelin 2 (also known as SM22a homologue), we focused our analysis on these two transcripts. cDNAs derived from the sham-operated animals and animals sacrificed at 2- and 8-day interval following ligation were used for RT-PCR. The mRNA levels were normalized to 18S rRNA and expressed as fold induction compared to sham-operated and ligated animals, (Figure 3). By Real Time PCR, it is found that although transgelin 1 and transgelin 2 are both regulated at transcriptional level and therefore are increased compared to the sham-operated animals, they have different kinetics as far as their mRNA expression is concerned. Transgelin 1 has a small increase on day 2 and increased further on day 8, while transgelin 2 seems to have a peak on day 2. Observed changes were statistically significant.

### Mapping of Transgelin Up-regulation in Specific Renal Cell Types

**Figure 4:**
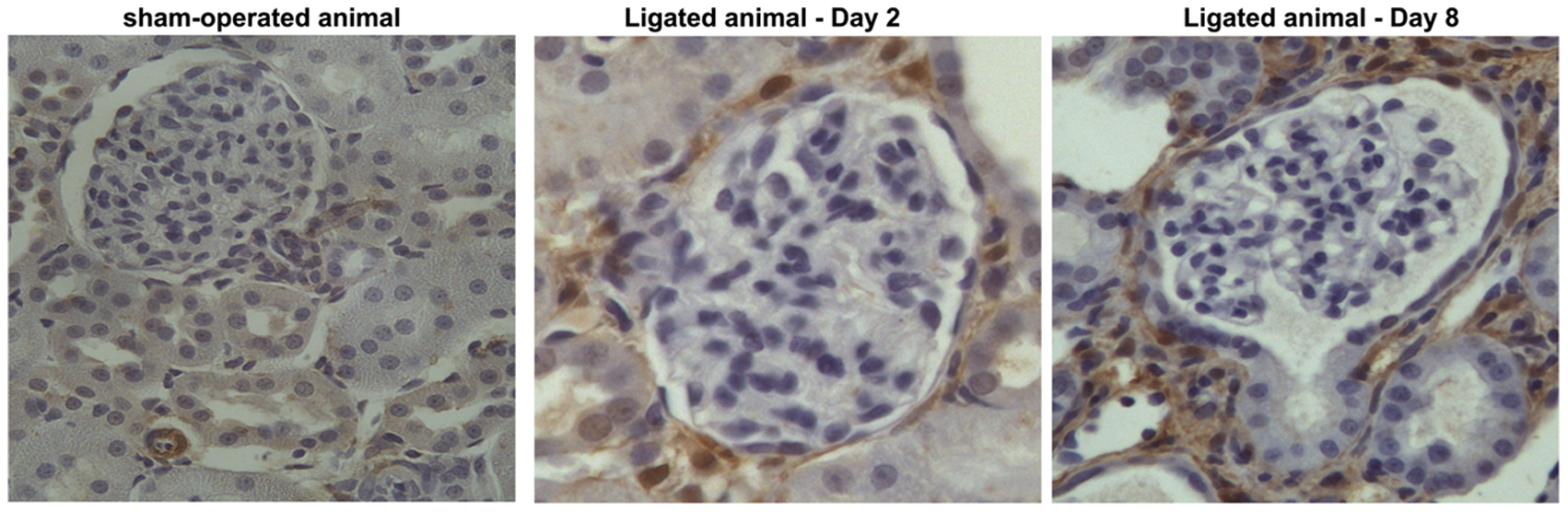
Localization of transgelin in rat kidney sections of the UUO model. Prominent staining is observed around glomeruli; it is also seen between tubules.

Since biochemical data both at the protein level and the mRNA level supported the finding that transgelin is up-regulated during the development of fibrosis, we next focused on identifying which cell types in the renal parenchyma were responsible for this phenomenon, by performing immunohistochemical studies. Sections from control and ligated animals were processed using the immunoperoxidase method. The results, shown in Figure 4, demonstrate that although transgelin exhibits a relatively low level of expression in control (sham-operated) animals, the expression of transgelin is up-regulated already at the 2-day and even more at the 8-day interval. In normal rat kidney (Figure 4A), transgelin is expressed in the smooth muscle cells of blood vessels. In ligated animals sacrificed 2 days (Figure 4B) after ligation transgelin is already present in cells that surround the glomeruli. In the ligated animals sacrificed 8 days after ligation (Figure 4C), transgelin has extended expression and it is found in the interstitial space as well as in cells that surround the glomeruli, just outside Bowman’s capsule. These periglomerular transgelin positive cells appear as spindle-like cells.

**Figure 5:**
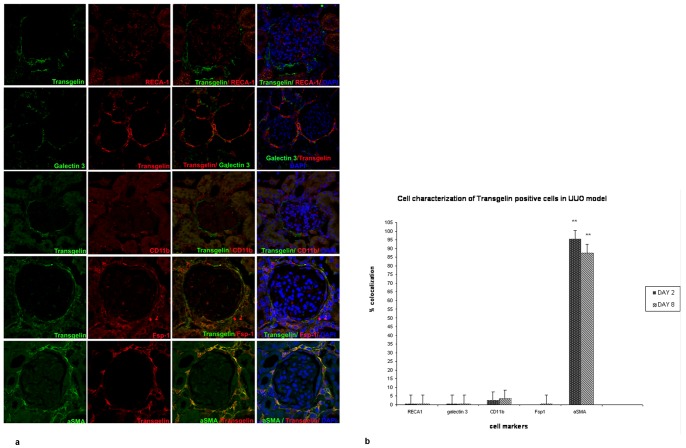
Cellular specificity of transgelin expression. a) Characterization of transgelin positive cells in the UUO model. Transgelin is not co-localized with any marker of different cell types except for α-SMA. b) Quantification of transgelin’s colocalization with markers of various cell types (Analysis by Image J Fiji). The asterisks denote statistical significance at a level of p<0.01.

We then proceeded to more specifically characterize these cells. The results are shown in figure 5A (a composite of representative confocal microscopy images) and are further quantified in figure 5B. In order to fully characterize the transgelin positive cells, double immunofluorescence experiments were performed for transgelin and a variety of markers of different cell types such as RECA-1 (rat endothelial cell marker), galectin-3 (epithelial and cells of the immune system marker), CD11b (dendritic cells and macrophage marker), Fsp1 (subtype of fibroblasts and cells of the immune system marker) and α-SMA (a marker for activated fibroblasts). No colocalization was found between transgelin and RECA-1, galectin-3, CD11b and Fsp-1 and therefore transgelin positive cells may not be endothelial cells, epithelial and cells of the immune system. On the contrary, based on the extensive statistically significant colocalization with α-SMA (Figure 5A & B), it can be concluded that transgelin positive cells may constitute a subpopulation of activated fibroblasts. The colocalization was calculated as it is described in the ‘material and methods’ section by ‘Image J Fiji’.

### Intracellular Localization of Transgelin

**Figure 6:**
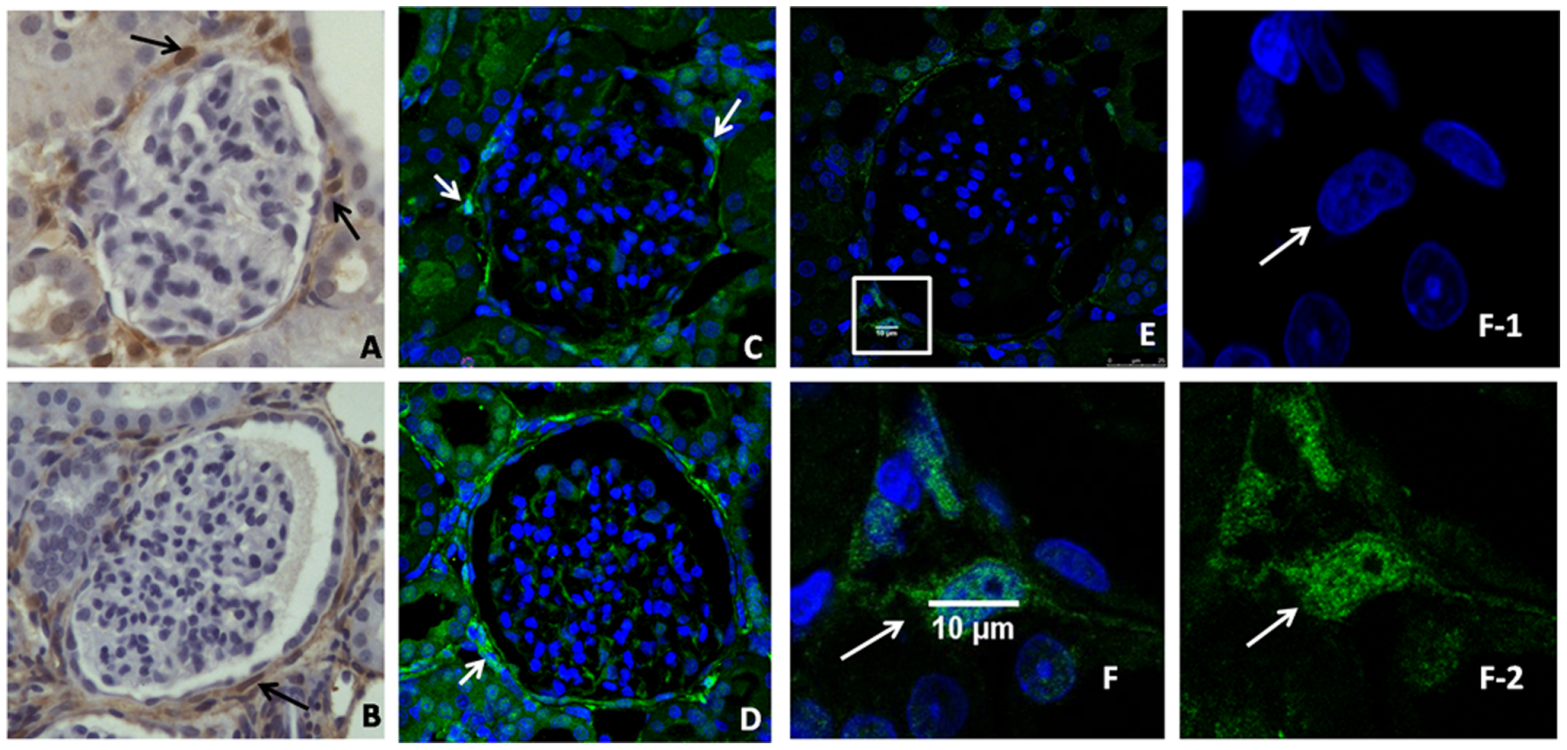
Nuclear localization of transgelin in the UUO model. Immunohistochemistry for transgelin in the ligated animals sacrificed after 2 days (A) and 8 days (B) of ligation. Immunofluorescence for transgelin in the ligated animals sacrificed after 2 days (C) and 8 days (D) after ligation. In 6E, a selected glomerulus under a 63x/1.4NA lens of confocal microscopy is seen, where transgelin is represented with green and the DAPI stains the nuclei (blue). In 6F (which splits in F1- transgelin/green chromophore and F2- DAPI/blue chromophore), a magnified area from the glomerulus of 6E is represented.

During our observations, we have noted that transgelin was as expected localized in the cytoplasm of the cells. However, we often have encountered images where transgelin was also localized in the nucleus. Figure 6 provides examples both with immunocytochemistry (Figure 6 A and B) and with immunofluorescence (Figure 6C and D) where transgelin is present in the nucleus. In particular, in Figure 6E and 6F, the intracellular localization is verified by confocal microscopy with the use of 63x lens in order to achieve high magnification and the thinnest optical section possible. It can be easily seen, that the chromophore for the antibody of transgelin is colocalized with DAPI, which stains nuclei. Therefore, transgelin was found inside the nucleus. As it is discussed in ‘Material and Methods’, the negative controls were samples which were incubated only with secondary antibody. These sections did not appear to exhibit any staining for transgelin (Figure S2 in File S1).

### Promoter Analysis of Transgelin

**Figure 7:**
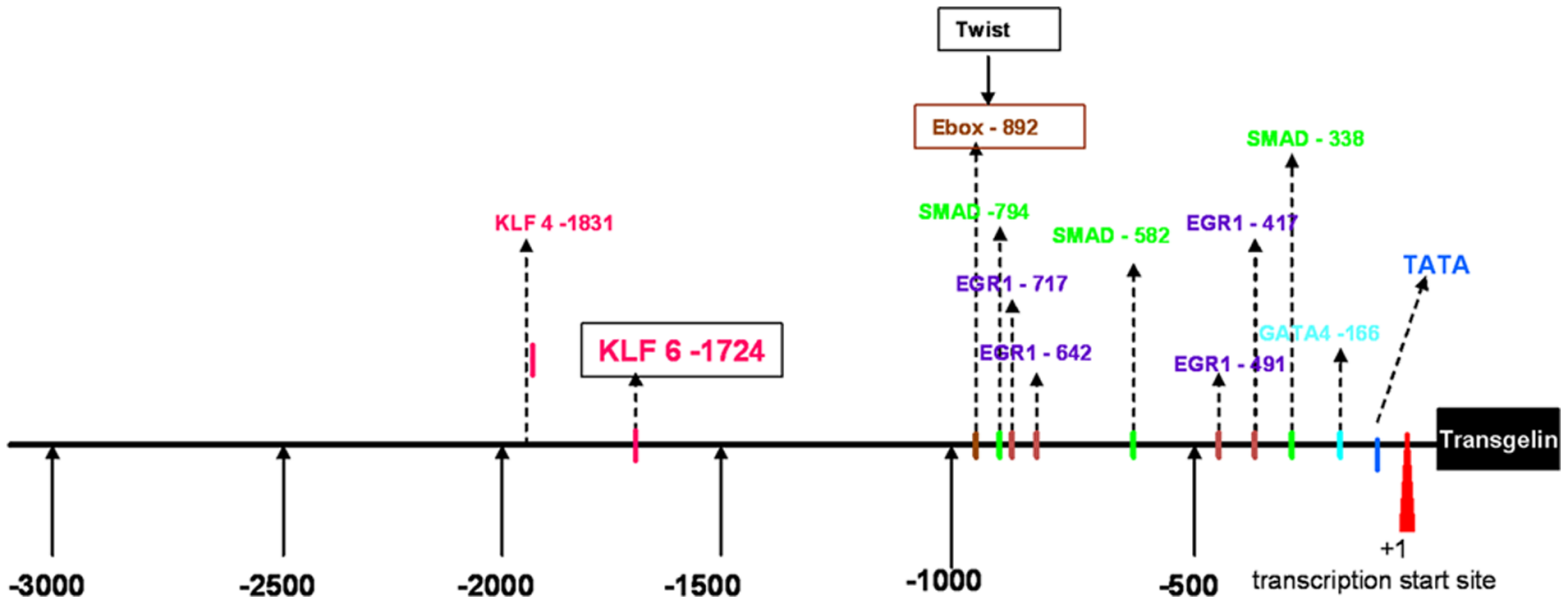
Schematic representation of transgelin’s promoter. The position of acceptor sites for transcription factors binding is shown.

In order to study the mechanism of transgelin overexpression in the fibrotic rat model, we proceeded to promoter analysis of transgelin. By this analysis, we investigated with Genomatix many transcription factors that have putative binding sites on the transgelin promoter sequence (Figure 7). Many of these transcription factors have been implicated with fibrosis.

In our experimental model of rat UUO, we tested the co-distribution of transgelin and a variety of transcription factors such as pSMAD2, pSMAD3, pSTAT3, b-catenin, EGR1, Msx1–2, Nkx2.5 and Twist (data not shown). By our technique, we found no colocalization of transgelin with any of the above transcription factors. Exception was Kruppel-like factor 6 (KLF-6), a transcription factor with a putative binding site on transgelin promoter (Figure 7) and more specifically on the promoter of the gene encoding transgelin 1 (but not transgelin 2).

**Figure 8:**
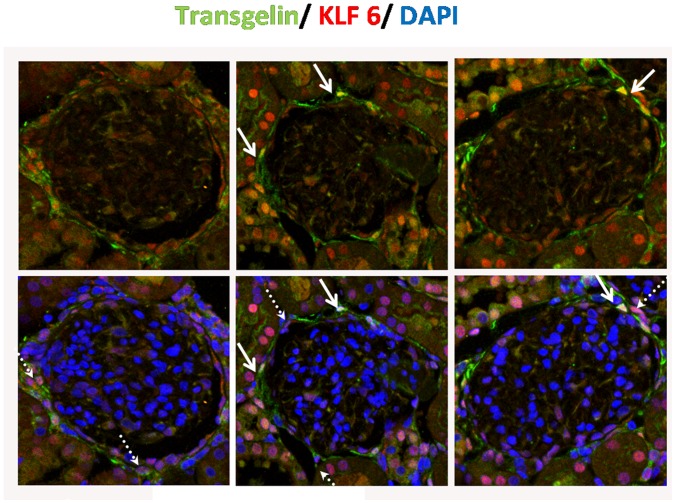
Co-expression of KLF6 and transgelin. Double Immunofluorescence for KLF6 (red) and transgelin (green) in rat sacrificed 2 days after ureteric ligation. The white arrow indicates cells that express KLF-6 and transgelin in their nuclei. The white dashed arrow indicates cells that express KLF-6 in their nuclei and transgelin in their cytoplasm.

Experiments of double immunofluorescence demonstrated that in normal animals KLF6 is localized in the nuclei of proximal tubules (data not shown). KLF-6 is dramatically increased on day 2 after ligation and it is present in cells that surround Bowman’s capsule and some of them colocalized with transgelin which could be either nuclear or cytoplasmic or both (Figure 8). Moreover, KLF-6 is colocalized with aSMA in the same model (data not shown).

Therefore, these morphological observations suggest that KLF-6 may be involved in transgelin up-regulation.

### Model of Hypertension vs Unilateral Ureteral Obstruction Model

**Figure 9:**
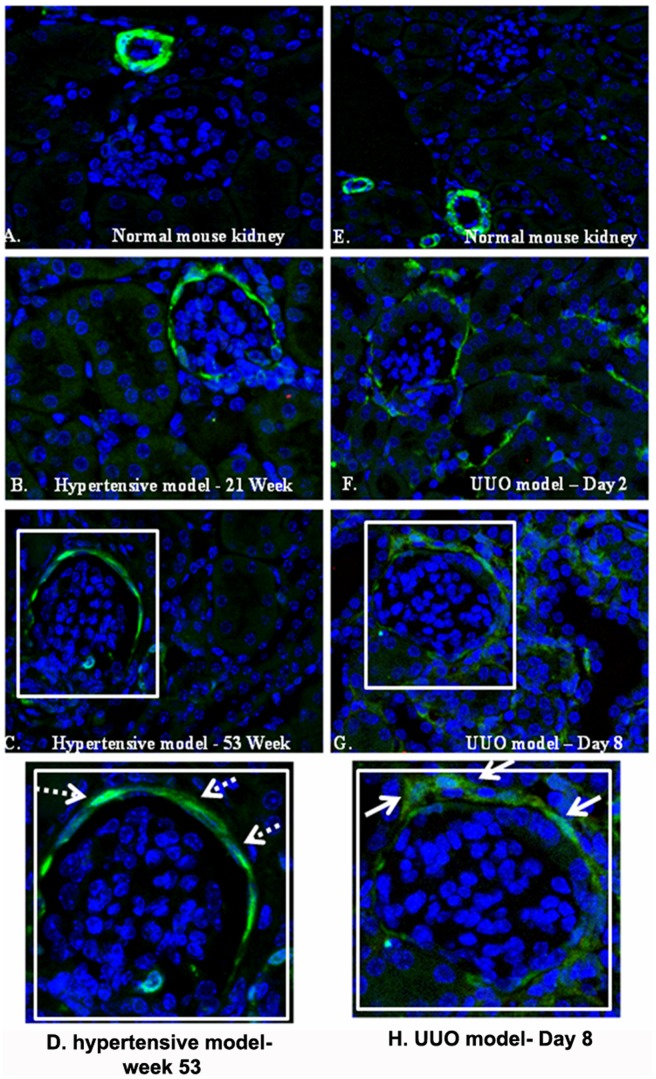
Immunofluorescence for transgelin in the mouse hypertension model vs in the UUO model. Contrary to the UUO model, in the hypertension model there are no transgelin-positive cells in periglomerular areas.

In order to explore whether the expression of transgelin in periglomerular fibroblasts represents a general feature of renal pathology during the development of fibrosis, we decided to compare the UUO model and the hypertension nephropathy model in mice. In Figure 9, we show representative fields of renal parenchyma having selected equivalent time intervals for the two models used. In UUO, day 2 post-ligation represents an early stage, where fibrotic tissue has not been developed (Figure 9F); in the hypertensive model, week 21 also represents an early stage where no fibrotic tissue accumulation has been recorded (Figure 9B). Next, in UUO, day 8 post-ligation is characterized by the development of fibrotic tissue, especially around glomeruli (Figure 9G) and in the hypertensive model, week 53 also represents a stage where fibrosis is observed (Figure 9C). It can be seen in the magnified areas of figure 9 that in the case of UUO (Figure 9H) there are several periglomerular cells positive for transgelin, whereas in the hypertensive model (Figure 9D), there are only transgelin positive cells inside the glomerulus, especially in the area of parietal epithelial cells, but no such staining is observed outside the glomerulus. These findings suggest that the ‘activation’ of periglomerular fibroblasts is not a general characteristic of fibrotic processes but rather a specific one related to the ligation model.

In order to get a general assessment of the transcription of transgelin in this model, the model was analyzed with the technique of RT-PCR, in order to complement the double immunofluorescence studies described above. The RT-PCR data showed a relative small up-regulation of the transgelin 1 transcript at all time intervals examined, but no significant change in the transcript of transgelin 2 (Figure S1 in File S1). Especially at the 53 week interval, where fibrotic tissue has started to accumulate, both transgelin transcripts demonstrate minimal alteration compared to age-matched control animals.

## Discussion

In this report, using the unilateral ureteric obstruction model for renal fibrosis in rodents, we focus on the expression of transgelin. We confirm proteomic analysis data and establish that expression of transgelin is up-regulated both in early and late time intervals. We establish that over-expression of transgelin is confined, especially at early time intervals in periglomerular “activated” fibroblasts and we demonstrate that this cell type is characteristic of the UUO model and is not “activated” in the hypertension-induced renal fibrosis.

To our knowledge, this is the first study focusing on the alterations in transgelin expression in obstructive nephropathy. Several studies have observed transgelin upregulation in other animal models: in the anti-GBM nephritis animal model in parietal epithelial cells of Bowman’s capsule [6], [9], [17], in the 5/6 nephrectomy animal model in interstitial fibroblasts and glomerular epithelial cells [7], in the puromycin aminonucleoside nephrosis animal model only in glomerular epithelial cells [7], in the adriamycin nephropathy and passive nephrotoxic nephritis animal models also in glomerular epithelial cells [8], [17], [18], in anti-Thy-1 nephritis animal model in mesangial cells [19]. Contrary to these studies, our results suggest that in obstructive nephropathy the first cell type to demonstrate transgelin up-regulation is the “periglomerular fibroblast”, whereas interstitial peritubular fibroblasts also show transgelin up-regulation especially at later stages. These data, taken together, suggest that depending on the animal model used, different cell types begin to demonstrate transgelin up-regulation. This conclusion is also supported by our findings, when we compare the UUO model with the hypertension-induced fibrosis in the animal model where renin is overexpressed [14].

The data produced in several animal models suggest cell-type specific up-regulation of transgelin at early time intervals, whereas as the time and the anatomical damage progresses, more cell types are prone to over-express transgelin. This is in agreement with our observations in human biopsy material, where we have noticed in many nephropathies of different etiology, many renal cell types over-expressing transgelin [10].

An interesting observation in our studies is the recorded intracellular localization of transgelin. In many instances, transgelin was observed not only in the cytoplasmic compartment, but also inside the nuclear compartment. To our knowledge, only one previous report exist where transgelin exhibited nuclear localization: it was observed in the nucleus of embryonic rat heart and isolated nuclei from cultured heart ventricular cells, examined by 2-D gel electrophoresis and mass spectrometry analysis [20]. Two important questions are generated from these observations: first, how transgelin is transported inside the nucleus, since it does not carry any nuclear localization signal in its sequence and second, what is the functional significance of the presence of transgelin inside the nucleus. More specifically it would be interesting to explore whether nuclear transgelin has a mere structural role or whether it may have any regulatory function.

Regarding the mechanism(s) involved in transgelin up-regulation, it is expected that several overlapping signaling pathways may be involved in this phenomenon. Studying the conserved transcription factor binding motifs on the transgelin promoter, it drew our attention the existence of a KLF-6 binding site. Physical stimuli (like electric field application) led dermal fibroblasts to overexpress KLF-6, as seen in microarray analysis [21]. During liver fibrosis, hepatic stellate cells (which can be considered as equivalent to fibroblasts) showed induction of KLF-6 induction at the mRNA and the protein level [22]. KLF-6 has been proposed as a TGF-beta promoter transactivator [23], [24]. Finally, in the animal model of renal ischemia-reperfusion, KLF-6 gene is among the few genes where transcription is increased more than 10-fold [25].

These observations, combined with the data presented in this work suggest a possible involvement of KLF-6 in renal pathology, worth further investigation in the future.

## Supporting Information

File S1 **Methodology of 2D gel electrophoresis for obtaining comparisons of renal parenchyma alterations in the UUO model.** Figure S1, Expression of transgelin 1 and transgelin 2 mRNAs in the hypertensive model. Real Time PCR was performed at the different time intervals indicated with the appropriate primers. The asterisk denotes statistical significance at a level of p<0.05. Figure S2, The specificity of nuclear staining of Transgelin. Figure 2A–C shows the staining of the negative control section of a ligated animal. The DAPI is shown in blue (2A), the staining of the secondary Cy2 anti-rabbit antibody is shown in figure 2B and the colocalization of them is presented in figure 2C. Figure 2D–F shows the staining of transgelin in a ligated animal. In 2D, the DAPI is shown in blue, in 2E the nuclear staining of transgelin is presented and in 2F, the colocalization of DAPI with transgelin.
(DOC)
