# Supplementary material for: Transgelin Up-Regulation in Obstructive Nephropathy
Source: PLoS One. 2013 Jun 26;8(6):e66887. doi: 10.1371/journal.pone.0066887 (PMC3694161; doi:10.1371/journal.pone.0066887)
Supplement: File S1 — Methodology of 2D gel electrophoresis for obtaining comparisons of renal parenchyma alterations in the UUO model. Figure S1, Expression of transgelin 1 and transgelin 2 mRNAs in the hypertensive model. Real Time PCR was performed at the different time intervals indicated with the appropriate primers. The asterisk denotes statistical significance at a level of p<0.05. Figure S2, The specificity of nuclear staining of Transgelin. Figure 2A–C shows the staining of the negative control section of a ligated animal. The DAPI is shown in blue (2A), the staining of the secondary Cy2 anti-rabbit antibody is shown in figure 2B and the colocalization of them is presented in figure 2C. Figure 2D–F shows the staining of transgelin in a ligated animal. In 2D, the DAPI is shown in blue, in 2E the nuclear staining of transgelin is presented and in 2F, the colocalization of DAPI with transgelin. (DOC) [file pone.0066887.s001.doc]

# SUPPLEMENTAL MATERIAL

# Methodology of 2D gel electrophoresis for obtaining comparisons of renal parenchyma alterations in the UUO model

# As it was mentioned in the ‘Material and Methods’ section, the proteomic analysis in UUO model was performed in the past in our lab and it has been published by Kypreou et al (Proteomics, 2008, 8: 2407-2419). In details, samples from kidney cortex containing 1000 mg of protein were extracted with 250 mL IEF sample buffer (7M urea, 2M thiourea, 50 mMTris-HCl, 2% CHAPS, 0.4% dithioerythritol (DTE), 0.01% bromophenol blue, 10 mL/mL from a mixture of protein inhibitors (Roche Diagnostics), pH 7.5). Samples were then applied onto nonlinear 17 cm strips of pI 3–10 (BioRad) in IEF cell focusing trays. Strips were rehydrated in 8 M urea, 0.4% DTE, 2% CHAPS, 0.2% carrier (3.5–10) ampholytes, 0.08% bromophenol blue overnight at room temperature. Sample application was performed using the cup loading method at the basic and acidic ends of the strip. After sample loading IEF took place for about 95 000– 100 000 V/h (250 V for 30 min, linear gradient 0–5000 V in 12 h, constant at 5000 V for 16 h and finally at 500 V for conservation). As soon as IEF was complete strips were incubated for 15 min at room temperature with equilibration solution I (6M urea, 50 mM Tris, 30% glycerol, 2% SDS, 30 mM DTE) followed by another 15 min with equilibration solution II (6 M urea, 50 mM Tris, 30% glycerol, 2% SDS, 136 mM iodoacetamide). The equilibrated strips were thenapplied on top of 12.5% polyacrylamide gels, fixed with molten agarose solution (0.5% agarose in Tris–glycine–SDS buffer) and run overnight at 10 mA/gel. Proteins were fixed with 50% methanol and 5% phosphoric acid for 2 h at room temperature. 2-D gels were stained with colloidal CBB for 24 h and destained with ultra pure water. Gels were scanned with a GS-800 calibrated densitometer and analyzed with PD Quest 7.2.0 image processing software (BioRad). Gel images from each group were edited and spots were matched manually. A unique identification number was assigned to matching spots on different gels. Normalization of the spot intensities was conducted according to the total OD in the gel. The stained protein spots of interest were excised manually from the gels and placed into 96-well microtiter plates. The excised spots were destained for 30 min at room temperature in 150 mL destaining solution per spot (50 mM ammonium hydrogen carbonate, 30% ACN), washed with ultra pure water (150 mL per spot) and dried in a vacuum centrifuge. Each dried spot was digested with 50 mg trypsin in 1 mM ammonium bicarbonate overnight at room temperature.Tryptic digests were extracted with 10 mL per spot of extraction solution (50% ACN, 0.1% TFA), for 20 min at room temperature. Peptide mixtures were analyzed with a MALDI-TOF/TOF mass spectrometer (Ultralfex II MALDITOF/ TOF-MS, Brucker Daltonics, Bremen, Germany). Peak lists were created with Flexanalysis v2.2 software (Brucker Daltonics). Peptide matching and protein searches were performed automatically by the use of MASCOT Software (Matrix Sciences, London, UK). For peptide identification, monoisotopic masses were used and a mass tolerance of 0.0025% (25 ppm) was allowed. Cysteine carbamidomethylation and methionine oxidation were set as fixed and variable modifications, respectively. One miscleavage was allowed. The peptide masses were compared with the theoretical peptide masses of all available proteins from Rattus Novergicus using Swiss-Prot database. The probability score with p < 0.05 identified by the software was used as the criterion for the affirmative protein identification.

# RT-PCR in the hypertensive model

# In the case of the hypertensive model, the extended time interval of the experiment (1 year) is accompanied by ageing of the animals. Therefore, in the relative RT-PCR experiments, every time interval should be studied in comparison with the equivalent control of the same age. The methodology used (described also in the section of Materials and Methods) is as follows:

# Primers were designed to amplify: 122bp of mouse transgelin 1, forward, 5’ AAGCCTTCTCTGCCTCAACAT 3’, reverse, 5’ CAATCCACTCCACTAGTCGCT 3’, 103bp of mouse transgelin 2, forward, 5’ TGTGGATCTCTGGGAAGGAAAG 3’, reverse, 5’ ATCCCCAGAGAAGAGCCCAT 3’. Primers were also designed to amplify 116bp of hypoxanthine guanine phosphoribosyl transferase (Hprt) gene, forward, 5’GGAGCGGTAGCACCTCCT 3’, reverse, 5’ CTGGTTCATCATCGCTAATCAC 3’.

Data were expressed as the ratio of transgelin 1 and transgelin 2 to the reference gene (HPRT) in the hypertensive model at several time points (9, 12, 21 and 53 weeks) both in wild-type and hypertensive animals.


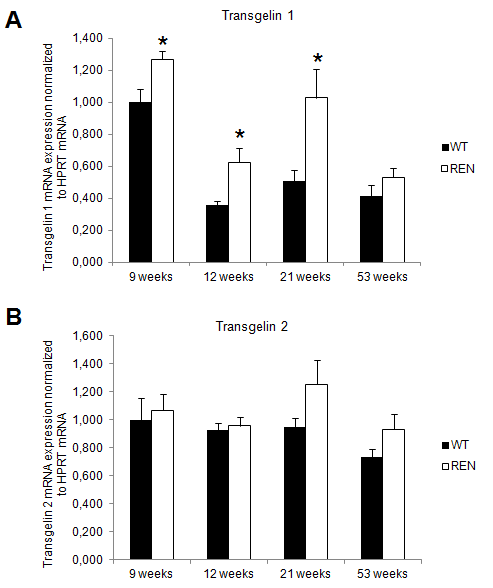


**Figure S1 of Supplemental material**: Expression of transgelin 1 and transgelin 2 in the hypertensive model. The asterisk denotes statistical significance at a level of p<0.05.

**Intracellular Localization of Transgelin**


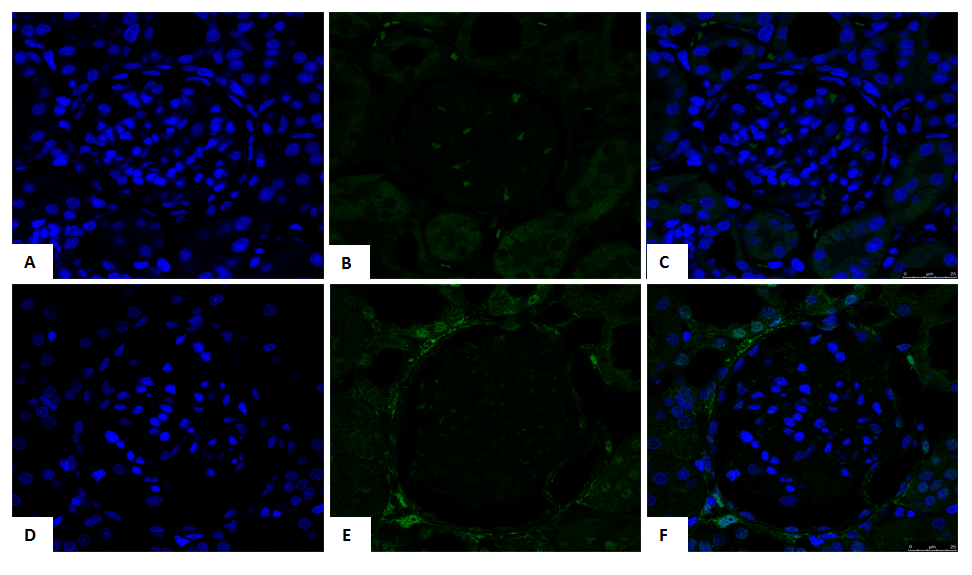


**Figure S2 of Supplemental material**: The specificity of nuclear staining of Transgelin.

Figure 2A-C shows the staining of the negative control section of a ligated animal. The DAPI is shown in blue (2A), the staining of the secondary Cy2 anti-rabbit antibody is shown in figure 2B and the colocalization of them is presented in figure 2C.

Figure 2D-F shows the staining of transgelin in a ligated animal. In 2D, the DAPI is shown in blue, in 2E the nuclear staining of transgelin is presented and in 2F, the colocalization of DAPI with transgelin.
